# Preparation and Gas-Sensitive Properties of Square–Star-Shaped Leaf-Like BiVO_4_ Nanomaterials

**DOI:** 10.3390/nano15020127

**Published:** 2025-01-16

**Authors:** Jin Liu, Mengdi Yang, Yuanyuan Lv, Yixin Gao, Danyang Bai, Neng Li, Haoru Guo, Anyi Wang

**Affiliations:** School of Communication and Information Engineering, Xi’an University of Science and Technology, Xi’an 710054, China

**Keywords:** BiVO_4_ nanomaterials, hydrothermal method, gas-sensing performance, gas sensor, ammonia

## Abstract

In this study, square–star-shaped leaf-like BiVO_4_ nanomaterials were successfully synthesized using a conventional hydrothermal method. The microstructure, elemental composition, and gas-sensing performance of the materials were thoroughly investigated. Morphological analysis revealed that BiVO_4_ prepared at different reaction temperatures exhibited square–star-shaped leaf-like structures, with the most regular and dense structures formed at 150 °C, exhibiting a large specific surface area of 2.84 m^2^/g. The response performance of the BiVO_4_ gas sensors to different target gases was evaluated, and the results showed that the prepared BiVO_4_ gas sensor exhibited a strong response to NH_3_. At the optimal operating temperature of 300 °C, its sensitivity to 5 ppm NH_3_ reached 13.3, with a response time of 28 s and a recovery time of 16 s. Moreover, the gas sensor exhibited excellent repeatability and anti-interference performance. These findings indicate that square–star-shaped leaf-like BiVO_4_ holds great potential in environmental monitoring and industrial safety detection, offering new insights for the development of high-performance gas sensors.

## 1. Introduction

With the continuous advancement of modern society and the rapid development of science and technology, significant progress has been made to benefit humanity. However, these advancements have also resulted in the discharge of waste gases and wastewater, leading to severe environmental challenges, one of the most pressing being air pollution. Air pollution occurs when harmful substances in the atmosphere exceed safe concentrations over a prolonged duration, causing adverse effects on human health and the environment [1,2,3]. This issue has caused significant harm for both humans and ecosystems. To safeguard public health and the environment, it is essential not only to implement measures to reduce air pollution but also to monitor harmful gases in the atmosphere effectively.

BiVO_4_ is an important functional oxide material characterized by its orthorhombic crystal structure and high symmetry [4]. Each BiVO_4_ molecule is composed of one vanadium atom, one bismuth atom and four oxygen atoms. The lattice structure alternates between vanadium–oxygen octahedra and bismuth–oxygen tetrahedra. In the lattice, vanadium ions are covalently bonded to oxygen atoms, while bismuth ions are surrounded by oxygen atoms, forming bismuth–oxygen tetrahedra [5,6,7]. These structural characteristics endow BiVO_4_ with unique properties, including a high capacity for Redox reactions and excellent electrical conductivity, making it highly suitable for gas-sensing applications [8,9].

BiVO_4_ is particularly promising for gas sensor development, especially in detecting harmful gases such as carbon monoxide, sulfur dioxide, nitrogen dioxide, and methane [10]. By controlling its morphology, structure and composition, its sensitivity to specific gases can be regulated, enabling the creation of highly sensitive and selective gas sensors [11]. These sensors have significant applications in environmental monitoring for the measurement harmful gas concentrations in the air, ensuring the safety of living environments, and preventing industrial accidents to enhance production safety [12].

Rabchinskii and Butko et al. [13,14,15,16,17] investigated a multi-sensor array based on hole-matrixed carbonylated (C-ny) graphene films for gas sensing at room temperature. The study demonstrated that C-ny graphene exhibited significantly better detection performance for methanol and ethanol at room temperature compared to pristine graphene, with higher sensitivity and selectivity. Experiments showed that the sensor’s minimum detection limits for methanol and ethanol were 0.05 ppm and 1.62 ppm, respectively, enabling the detection of trace concentrations. Additionally, the sensor array exhibited excellent long-term stability, with the porous structure of C-ny graphene and the abundance of carbonyl groups were identified as the key factors enhancing its gas response performance. Kus et al. [18] designed and fabricated a dual-channel gas sensor based on few-layer MoS_2_ for the detection of volatile organic compounds (VOCs). The sensor platform consisted of two parallel sensors made from the same material, with one part kept in its original state (electron-dominated sensor) and the other subjected to UV-O_3_ treatment to form a hole-dominated sensor. Experimental results showed that the original MoS_2_ sensor exhibited responses of 18%, 3.5%, and 49% to 3000 ppm ethanol, acetone, and toluene, respectively, while the UV-O_3_-treated MoS_2_ sensor exhibited responses of 13.4%, 3.1%, and 6.7%, respectively. This dual-channel sensor system demonstrated superior selectivity for toluene, with a selectivity that was seven times higher than that for ethanol and acetone. Rabchinskii et al. [19] successfully carboxylated graphene through a photochemical method, introducing up to 9.5 at% carboxyl groups while forming a nanopore array, yet retaining the layered and planar structure of graphene. The carboxylated graphene exhibited excellent gas-sensing performance at room temperature, enabling the efficient detection and differentiation of various alcohols, acetone, and ammonia. Under humid conditions, ammonia exhibited an opposite chemiresistive response, with the mechanism elucidated through density functional theory (DFT) calculations, highlighting the effects of adsorbed ammonia and water on the Fermi level and charge transfer. 

Bai et al. [20] prepared nanoplates with a thickness of about 10 nm and a width of about 1 μm using a hydrothermal method with the addition of ethylenediaminetetraacetic acid (EDTA). The gas-sensitive test results showed that the nanoplates had high selectivity for NO_2_ and demonstrated a response value of 2.3 for 4 ppm NO_2_ at a low ambient temperature (60 °C). This was attributed to the small nanometer size, which increased the specific surface area, and the plate shape, which facilitated the rapid reaction of the gas, but the response/recovery time of the BiVO_4_ was long (40 s/70 s). Golmojdeh et al. [21] prepared pure BiVO_4_ and lanthanum-doped BiVO_4_ (La-BiVO_4_) using V_2_O_5_ and Bi(NO_3_)_3_ as raw materials through a precipitation method. They compared the gas sensitivity by testing their responses to different concentrations of ethanol gas, finding that La-BiVO_4_ exhibited higher sensitivity, primarily attributed to its smaller particles and the presence of tetragonal BiVO_4_. Yang et al. [22] prepared pure and Fe-Mo co-doped BiVO_4_ nanomaterials using the solution combustion synthesis (SCS) method. The gas-sensing test results showed that the Fe-Mo co-doped BiVO_4_ gas sensor exhibited an excellent response and sensitivity (−148.988 mV/decade) to low concentrations of NH_3_ (10–50 ppm). Additionally, Fe and Mo doping enhanced its selectivity for NH_3_. Wang et al. [23] synthesized BiVO_4_/Bi_4_V_2_O_11_ heterojunction mixed-potential NH_3_ sensor materials via the hydrothermal method, followed by self-phase separation treatment. Gas-sensing results indicated that this sensor exhibited the highest sensitivity and the lowest detection limit, with sensitivities of −6.9 and −38.2 mV/decade for NH_3_ concentrations of 25 ppm and 300 ppm, respectively. Furthermore, sensitivity tests showed that coexisting gases such as CO_2_, CH_4_, H_2_, and H_2_S caused response variations of −3.7%, −3.0%, 0.9%, and −6.8%, respectively, demonstrating good anti-interference performance. Meng et al. [24] synthesized flower-like Bi_0.95_Ni_0.05_VO_3.975_ using the hydrothermal method. By using Bi_0.95_Ni_0.05_VO_3.975_ as the sensing electrode and La_10_Si_5.5_Al_0.5_O_27_ as the electrolyte, they fabricated a mixed-potential NH_3_ sensor. Gas-sensing performance tests showed a response potential of 52.4 mV for 400 ppm NH_3_. Guan et al. [25] studied the laser-driven phase transition technology of two-dimensional transition metal dichalcogenides (TMDs) and reported a method to transform metallic 2M-WS_2_ into semiconducting 2H-WS_2_ using a femtosecond (fs) laser. The study also revealed that the laser-irradiated 2H-WS_2_ was rich in sulfur vacancies and exhibited excellent ammonia-sensing performance, with a detection limit below 0.1 ppm and rapid response/recovery times of 43/67 s at room temperature. This research provides a new strategy for the preparation of phase-selective homojunctions and the development of high-performance electronic devices. Chowdhury et al. [26] successfully synthesized a series of hierarchical three-dimensional (3D) manganese–cobalt BTC (Mn-CoBTC, BTC = benzene-1,3,5-tricarboxylate) metal–organic frameworks (MOFs) with controllable sub-units and internal structures without the need for templates, etchants, or high-pressure/high-temperature conditions. The study demonstrated that M2C-BTC exhibited excellent ammonia (NH_3_)-sensing performance, with a low detection limit (1.12 ppm), high selectivity (41%), and outstanding stability, showing only a 3.1% change in ΔF value after 5 months of testing. Quan et al. [27] developed a flexible gas sensor (MNPE–Ni–N–C/Ti_3_C_2_T_x_) based on Ni single-atom active sites (Ni–N–C) and 2D MXene nanosheets (Ti_3_C_2_T_x_), achieving highly sensitive, selective, and stable ammonia (NH_3_) detection. The sensor demonstrated a response value of 27.3% to 5 ppm NH_3_, with a theoretical detection limit as low as 12.1 ppb, performing 1.7 times better than traditional Au electrode sensors. Additionally, the sensor exhibited outstanding stability, with resistance and response values decreasing by only 3.9% and 9.0%, respectively, over 4 weeks, significantly outperforming Au electrode sensors. 

NH_3_ sensors based on BiVO_4_ and its doped or modified materials deserve attention mainly because of their high sensitivity and low detection limit, which enable the accurate detection of low concentrations of NH_3_. Additionally, they exhibit excellent selectivity and anti-interference performance. In industrial and environmental monitoring, these materials show broad application prospects for detecting low-concentration gases and operating in complex atmospheres, making them highly valuable for research.

Square–star-shaped leaf-like BiVO_4_ nanomaterials have significant potential for various applications, particularly in environmental monitoring, energy conversion, and related fields. As a sensitive material for gas sensor fabrication, these nanostructures enhance the adsorption and reaction of target gases, enabling the precise detection and monitoring of gas compositions. The real-time monitoring of gas compositions in environmental, industrial safety, and biomedical contexts is critically important. Thus, studying the gas-sensitive properties of square–star-shaped leaf-like BiVO_4_ nanomaterials is highly valuable for the development of high-performance gas sensors [28,29,30].

This study employs a conventional hydrothermal method to synthesize square–star-shaped leaf-like BiVO_4_ powder and investigates its gas-sensing properties. The unique morphology of this material increases its specific surface area, providing more active sites for gas molecules, enhancing the interaction between the gas and the material, accelerating reaction rates, and improving response times. Moreover, the increased number of reaction sites enhances the sensitivity of the material to low-concentration gases, enabling more efficient gas detection.

## 2. Experience Section

### 2.1. Chemical Reagents

Chemical reagents included bismuth nitrate pentahydrate (Bi(NO_3_)_3_·5H_2_O, 99% purity), sodium metavanadate (Na_3_VO_4_·12H_2_O, 99% purity), and absolute ethyl alcohol (C_2_H_6_O, 99% purity). All were purchased from Sinopharm Chemical Reagent Co., Ltd. (Ningbo Road, Shanghai, China).

### 2.2. Experimental Method

#### 2.2.1. Preparation of BiVO_4_ Powder

Bismuth nitrate (Bi(NO_3_)_3_·5H_2_O) was used as the bismuth source and sodium metavanadate (Na_3_VO_4_·12H_2_O) as the vanadium source to prepare BiVO_4_ powder materials. First, an appropriate amount of bismuth nitrate (Bi(NO_3_)_3_·5H_2_O) was added to deionized water and subjected to ultrasonic treatment for 30 min to ensure complete dissolution, resulting in a Bi(NO_3_)_3_ solution with a concentration of 5 mmol/L, while a sodium metavanadate solution with a concentration of 10 mmol/L was prepared in parallel. The [Bi^3+^]/[VO_4_^−^] molar ratio was maintained at 1:2 by adding the Bi(NO_3_)_3_ solution dropwise into the Na_3_VO_4_ solution under magnetic stirring, resulting in a yellow suspension. This yellow suspension was then transferred to a polytetrafluoroethylene high-pressure reactor, filled to about 70% of its capacity, and heated at suitable temperatures (140 °C, 150 °C, 160 °C, and 170 °C) for 8 h. After the reaction was complete, the mixture was allowed to cool naturally to room temperature. The resulting product was washed three times with deionized water and anhydrous ethanol. The washed samples were placed in a beaker, and the drying oven was set to 70 °C for a certain period to obtain the final product. The obtained products were labeled A1 (140 °C), A2 (150 °C), A3 (160 °C), and A4 (170 °C), according to their temperatures from low to high, for characterization and gas sensitivity testing. 

#### 2.2.2. Characterization

X-ray diffraction (XRD) was used to measure the crystal structure of the sample using a Cu-Kα radiation source (wavelength = 0.15406 nm) with a scanning angle range of 5° to 90°. The sample was prepared as a fine powder and placed on the sample holder. X-rays interacted with the sample, producing diffraction patterns. By analyzing the diffraction patterns, the phase composition and crystallinity of the sample were determined. By comparing the position and intensity of the diffraction peaks, the crystal structure and crystallinity of the sample could be identified. A scanning electron microscope (SEM, QUANTUM SEM 5000X + UltimMax 40e, Abingdon, UK) was used to observe the microstructure and lattice spacing of the prepared sample, equipped with an energy dispersive X-ray spectrometer (EDS) for the quantitative and distribution analysis of surface elements. The accelerating voltage was set at 10 kV with a resolution of 1 nm. The SEM was used to observe the microstructure and lattice structure of the sample surface, while the EDS provided qualitative and quantitative analysis of the surface elements and their distribution. Additionally, the Thermo SCIENTIFIC ESCALAB Xi+ X-ray (Thermo Fisher Scientific Inc., Waltham, MA, USA) photoelectron spectrometer (XPS) was employed to characterize the chemical states and binding energies of surface elements. A monochromatic aluminum target (Al-Kα) was used as the X-ray source, with a vacuum of 10^−9^ mbar and a resolution of 0.5 eV. Binding energy calibration was performed using the C1s peak (284.8 eV) as a reference. During the experiment, the sample was placed in a vacuum environment, and X-rays irradiated the sample surface, exciting photoelectrons, which were measured by the analyzer to obtain the XPS spectrum. By analyzing the shifts and shapes of characteristic peaks, the chemical states and binding energies of the surface elements were determined. The above-mentioned equipment and parameters ensured the accuracy and reliability of the experimental data, providing a solid foundation for the in-depth analysis of the sample’s structure and chemical properties. The specific surface area of the sample was measured using the BET method on a Micromeritics ASAP 2460 instrument (Shanghai, China). After degassing treatment, a nitrogen adsorption–desorption test was conducted under 77 K liquid nitrogen conditions using the fully automated BET surface area analyzer. Once the analysis was completed, the adsorption–desorption isotherm curve was obtained. By analyzing the adsorption isotherm of nitrogen (N_2_) on the surface of the material, the BET method was used to calculate the monolayer adsorption volume and further derive the specific surface area of the sample. 

#### 2.2.3. Fabrication and Testing of BiVO_4_ Sensors

**Figure 1:**
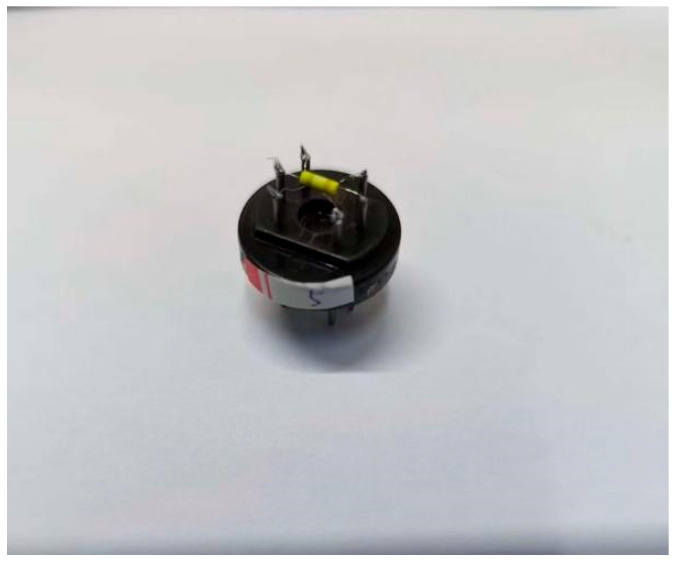
BiVO_4_ gas sensor.

The preparation of the BiVO_4_-based gas sensor is completed through the following steps: First, 1.0 g of BiVO_4_ powder is dispersed in a mixed solvent of 5 mL anhydrous ethanol and 5 mL deionized water to prepare a uniform viscous slurry. This slurry is evenly and smoothly coated onto an alumina (Al_2_O_3_) substrate, ensuring that the powder coating completely covers the electrodes. To guarantee the uniformity and reproducibility of the sensor’s performance, the coating thickness is adjusted to 10 μm. This is achieved through stepwise coating, where each thin layer is allowed to dry before applying the next, until the desired thickness is reached. Optical microscopy is used to confirm the coating thickness. Controlling the coating thickness is crucial to avoid excessive thickness, which increases resistance, or insufficient thickness, which reduces sensor sensitivity. The coated samples are dried at 100 °C for 2 h to remove the solvent, followed by calcination at 300 °C for 1 h to form a stable structure for the BiVO_4_-based gas sensor. The prepared BiVO_4_ gas sensor is shown in Figure 1 below.

After preparing the gas sensor, a WS-30A gas-sensitive test system was used to test the gas-sensing performance. In this experiment, ammonia (NH_3_) was selected as the target gas to evaluate the gas-sensing performance of the square–star-shaped leaf-like BiVO_4_ nanomaterial. Ammonia is a widely used industrial chemical and a common pollutant, making its detection crucial for environmental monitoring and industrial safety. The WS-30 A gas-sensing test system was employed to measure the sensor’s response to different concentrations of NH_3_ (0.1 ppm to 5 ppm) in order to evaluate its gas-sensing performance, stability, optimal operating temperature, and other factors. Sensitivity, as a key parameter for evaluating gas sensor performance, is defined as the ratio of the sensor’s current before and after exposure to the gas, expressed by the following formula:

(1) $$S=\frac{I_{g}}{I_{a}}$$

where *I_a_* represents the current of the sensor in air, and *I_g_* represents the current in the target gas environment (NH_3_). This experiment not only analyzed the sensitivity variation of the sensor through test data at different concentrations but also further examined the response and recovery times of the sensor to evaluate its dynamic response capability. In addition, the optimal operating temperature and stability of the sensor were tested to ensure reliable performance under different testing conditions. 

## 3. Results and Discussion

### 3.1. Characterization of BiVO_4_

**Figure 2:**
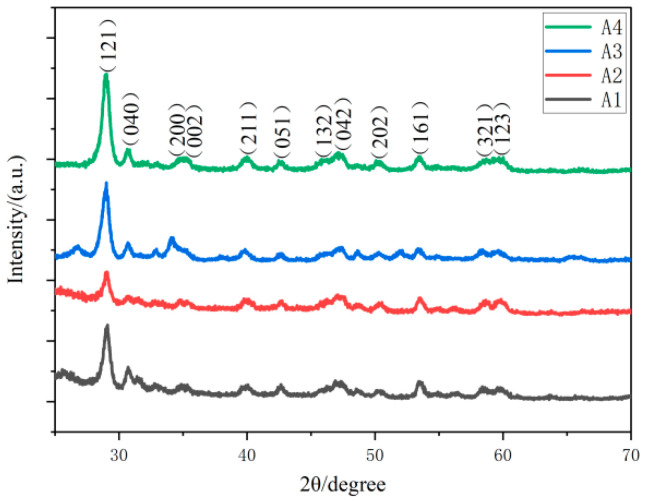
XRD patterns of BiVO_4_ samples prepared at different temperatures: A1 (140 °C), A2 (150 °C), A3 (160 °C), and A4 (170 °C).

The structure of the prepared BiVO_4_ powder material was tested and analyzed, and the results are shown in Figure 2. According to the spectrum, the diffraction peaks located at 28.98°, 30.64°, 34.8°, 35.18°, 40°, 42.46°, 45.82°, 47.08°, 50.08°, 53.4°, 58.7°, and 59.68° correspond to the (121), (040), (200), (002), (211), (051), (132), (042), (202), (161), (321), and (123) crystal planes of BiVO_4_, respectively, and all match well with the standard card for monoclinic BiVO_4_ (JCPDS 14-0688) [31]. Among these, the (121) crystal plane exhibits the strongest diffraction peak, indicating that the prepared BiVO_4_ has a significant preferential growth orientation in the (121) plane, which is thermodynamically more stable. Except for this, no impurities or secondary phases were observed in the spectrum, indicating that the synthesized samples A1, A2, A3, and A4 are pure BiVO_4_ with a monoclinic scheelite structure. 

**Figure 3:**
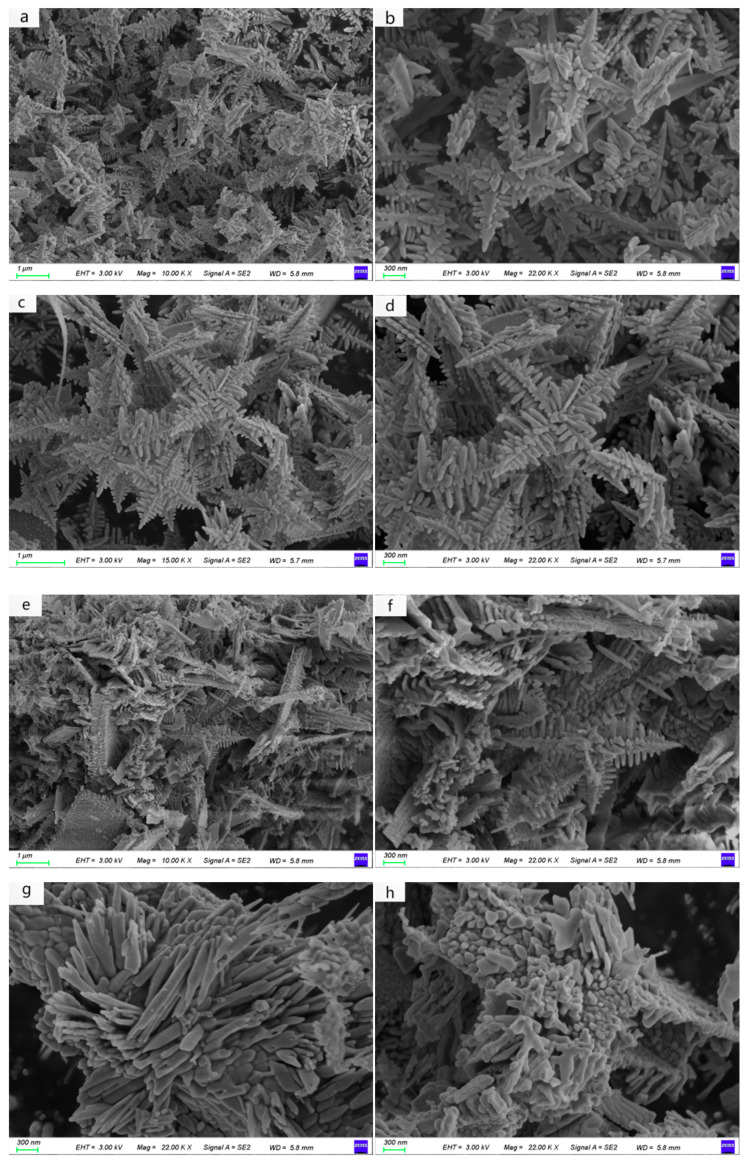
SEM images of BiVO_4_ samples prepared at different temperatures: (**a**,**b**) A1 (140 °C), (**c**,**d**) A2 (150 °C), (**e**,**f**) A3 (160 °C), and (**g**,**h**) A4 (170 °C).

The morphology of BiVO_4_ nanomaterials was characterized using field emission scanning electron microscopy (SEM), as shown in Figure 3. In Figure 3a,b, it can be clearly seen that at a reaction temperature of 140 °C, a square–star-shaped leaf-like structure is formed, with densely packed, regularly shaped leaves and a very small amount of rod-like structures. When the reaction temperature is increased to 150 °C, as shown in Figure 3c,d, the square–star-shaped leaf-like structure becomes more distinct and regular, with denser leaves and no other structures present. At a reaction temperature of 160 °C, as illustrated in Figure 3e,f, a small number of square–star-shaped leaf-like structures form, along with many irregular rod-like structures. When the temperature is further increased to 170 °C, as shown in Figure 3g,h, no square–star-shaped leaf-like structures are produced; instead, a large number of fine, rod-like structures aggregate together in an irregular arrangement. This suggests that excessively high temperatures are not conducive to the formation of the square–star-shaped leaf-like structure. 

**Figure 4:**
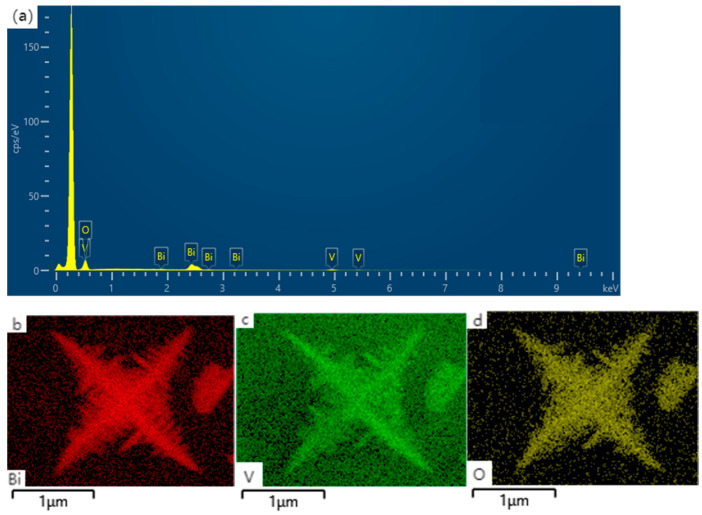
(**a**) EDS spectrum (**b**–**d**) mapping of the BiVO_4_ material with a square–star-shaped leaf-like structure prepared at 150 °C.

The surface elemental distribution of the prepared BiVO_4_ nanomaterials was analyzed using SEM-EDX, as shown in Figure 4 and Table 1. Energy dispersive X-ray analysis revealed the elemental distribution in the sample to be oxygen (O) at 77.1%, bismuth (Bi) at 12.5%, and vanadium (V) at 10.4%. Figure 4b–d shows the energy spectrum maps of the square–star-shaped leaf-like BiVO_4_ material synthesized at 150 °C, where Figure 4b represents the Bi element, Figure 4c represents the V element, and Figure 4d represents the O element. It can be observed that the Bi, O, and V elements are uniformly distributed and highly overlapped, indicating good chemical bonding between them. Furthermore, the absence of other elements in the image suggests that the prepared sample consists of BiVO_4_ nanomaterials, which is consistent with the results of XRD and SEM analyses. 

To determine the chemical states and electronic structure of surface elements in the square–star-shaped leaf-like BiVO_4_ nanomaterials synthesized at 150 °C, XPS was employed. During the test, an ESCALAB Scientific K-Alpha+ X-ray photoelectron spectrometer (XPS) was used, with the energy resolution set to 0.5 eV and a pass energy of 20 eV. In the data analysis process, Avantage (59922) software was used to subtract the background signal from the binding energy spectrum through a linear background correction method. Two background points were selected on the left and right sides of the target peak, and the software automatically fitted a linear background curve. This curve was then subtracted from the original spectrum to generate a corrected spectrum, eliminating the background influence caused by scattered electrons. The spectrum fitting was also performed using Avantage software. The process began with data preprocessing, including energy calibration using the C1s peak (284.8 eV) as a reference and selecting an appropriate linear background correction method to remove unrelated background signals. Subsequently, fitting peaks were added within the range of the target peak, and a peak shape model was selected. The mixing ratio and peak width were adjusted as needed. The peak positions were initialized based on theoretical binding energy values, and the peak intensities and widths were gradually optimized to achieve a good fitting result. After fitting, the quality of the fit was evaluated by checking the consistency of the fitted curve with the experimental data and examining the residual distribution. If necessary, parameters were adjusted or additional peaks were added to improve the fitting. Finally, the fitted spectrum data and graphs were exported, and key parameters such as peak positions, peak widths, and peak areas were recorded to ensure the accuracy and reproducibility of the data analysis. 

**Figure 5:**
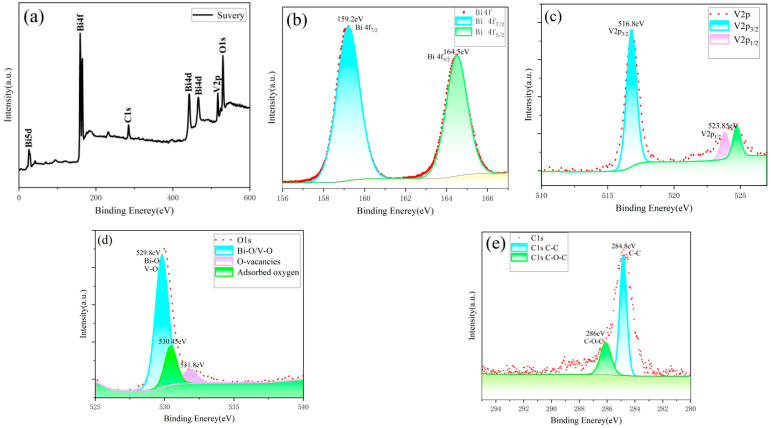
XPS spectra of the (**a**) survey, (**b**) Bi4f, (**c**) V2p, (**d**) O1s, and (**e**) C1s of BiVO_4_ nanomaterials with a square–star-shaped leaf-like structure prepared at 150 °C.

The test results are shown in Figure 5. In the survey spectrum of the BiVO_4_ nanomaterials with square–star-shaped leaf-like structures (Figure 5a), distinct characteristic peaks for Bi, V, O, and C were observed, indicating that the sample was primarily composed of Bi, O, and V. Aside from the standard C peak used for calibration, no impurities were detected [32]. Figure 5b presents the high-resolution spectrum of Bi4f, where the two main peaks are located at binding energies of 159.2 eV and 164.5 eV, corresponding to Bi 4f_7/2_ and Bi 4f_5/2_, respectively. This confirms that bismuth exists in the trivalent state, likely derived from bismuth compounds [33]. Figure 5c shows the high-resolution spectrum of V 2p, with peaks located at 516.8 eV (V2p_3/2_) and 523.85 eV (V2p_1/2_), indicating that vanadium is primarily in the pentavalent oxidation state, corresponding to the oxidation state of V in VO_4_^−^. These peak positions are consistent with literature reports, suggesting that the chemical environment of vanadium in the material is normal and that no significant lower-valence vanadium states (such as V^4+^ or V^3+^) are present. The peak area ratio of V 2p_3/2_ to V 2p_1/2_ is close to the theoretical value of 2:1, further confirming the accuracy of the fitting. This indicates that the sample effectively maintains the stability of pentavalent vanadium during the preparation process, with minimal influence of oxygen vacancies on the oxidation state of vanadium [34]. Figure 5d shows the high-resolution spectrum of O 1s. The peak at 529.8 eV corresponds to lattice oxygen (Bi–O and V–O) in metal oxides, reflecting the lattice integrity of the material. The peak at 530.45 eV is attributed to oxygen vacancies (O-vacancies), indicating the presence of non-stoichiometric oxygen species such as O^−^ and O_2_^−^, which are related to the redox properties of the sample and contribute to its enhanced gas-sensing performance. The peak at 531.8 eV corresponds to surface-adsorbed oxygen species (such as hydroxyl groups or molecular oxygen), which are closely associated with the surface chemical activity of the material [35]. Figure 5e presents the high-resolution spectrum of C 1s, with the peak located at a binding energy of 284.8 eV. Therefore, there is no need for the additional calibration of other XPS peaks for the elements. This is because the analysis was conducted using an ESCALAB Scientific K-Alpha+ X-ray photoelectron spectroscopy (XPS) instrument. The binding energy calibration used the C 1s peak at 284.8 eV on the sample surface as the reference, which is an internationally recognized standard for XPS calibration [36,37,38,39].

**Figure 6:**
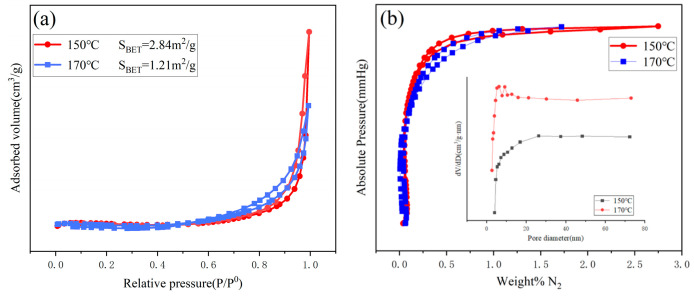
BET curves of BiVO_4_ nanomaterials prepared at 150 °C and 170 °C: (**a**) N_2_ adsorption–desorption isotherms and (**b**) pore size distribution curves and pressure isotherms.

The specific surface area of gas-sensitive materials is one of the key factors influencing their gas-sensing performance. The specific surface areas of BiVO_4_ nanomaterials prepared at 150 °C and 170 °C were tested, and the results are shown in Figure 6a,b and Table 2. Figure 6a shows that the N_2_ adsorption–desorption isotherms of both samples resemble Type IV isotherms according to the IUPAC classification, with H_3_-type hysteresis loops, indicating that the materials are predominantly mesoporous. Based on the BET model, the specific surface areas of BiVO_4_ nanomaterials prepared at 150 °C and 170 °C are 2.84 m^2^/g and 1.21 m^2^/g, respectively, with the 150 °C sample exhibiting a larger specific surface area and better gas-sensing performance (consistent with SEM analysis) [40]. Figure 6b presents the pore size distribution and pressure isotherms. According to the BJH model, the average pore sizes of the samples prepared at 150 °C and 170 °C are 17.76 nm and 42.09 nm, with pore volumes of 0.026 cm^3^/g and 0.021 cm^3^/g, respectively [41]. The comparison indicates that the 150 °C sample has a larger specific surface area, higher pore volume, and smaller average pore size, resulting in better gas-sensing performance than the 170 °C sample. 

### 3.2. Gas-Sensing Performance

**Figure 7:**
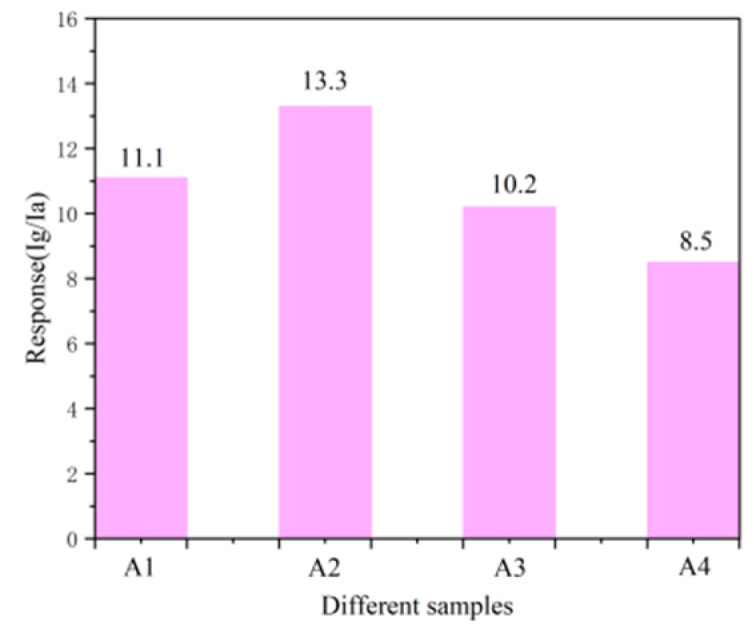
Gas-sensitive response of BiVO_4_ samples prepared at different temperatures to 5 ppmNH_3_ at 300 °C: A1 (140 °C), A2 (150 °C), A3 (160 °C), and A4 (170 °C).

The gas-sensing response of BiVO_4_ samples prepared at different temperatures to 5 ppm NH_3_ at 300 °C was tested, as shown in Figure 7. The gas-sensing responses of samples A1 (140 °C), A2 (150 °C), A3 (160 °C), and A4 (170 °C) were 11.1, 13.3, 10.2, and 8.5, respectively. It is clear that sample A2 exhibited the highest gas-sensing response, while sample A4 showed the lowest response. This trend aligns with the SEM results discussed earlier, indicating that the BiVO_4_ sample synthesized at 150 °C demonstrates optimal gas-sensing performance. Subsequently, further in-depth studies were conducted on the gas-sensing properties of the square–star-shaped leaf-like BiVO_4_ sample prepared at 150 °C (sample A2). 

**Figure 8:**
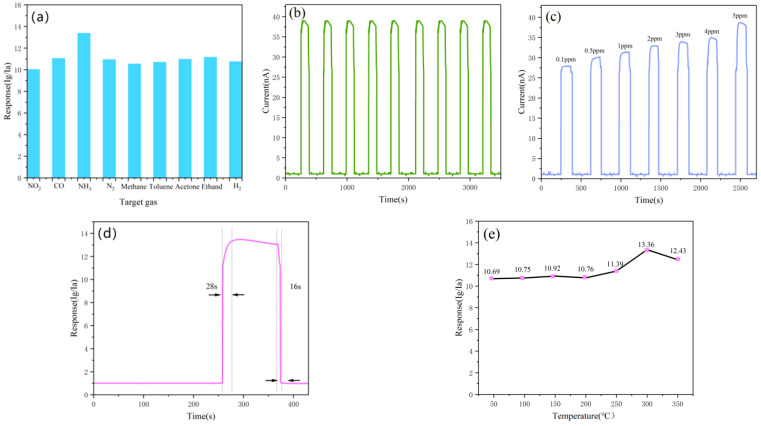
Gas-sensitive properties of the square–star-shaped leaf-like BiVO_4_ sample (A2) prepared at 150 °C. (**a**) Gas-sensitive response diagram to different gases at 300 °C. (**b**) Repeatability test diagram for 5 ppmNH_3_ at 300 °C. (**c**) Transient response curve to different concentrations of NH_3_ at 300 °C. (**d**) Response–recovery curve for 5 ppmNH_3_ at 300 °C. (**e**) Gas-sensitive response to 5 ppmNH_3_ at different operating temperatures.

The gas-sensing performance of the square–star-shaped leaf-like BiVO_4_ sample prepared at 150 °C was studied in greater depth. As shown in Figure 8a, the gas-sensitive properties of a square–star-shaped leaf-like BiVO_4_ sample prepared at 150 °C to different gases were tested. The test data showed that the sensor prepared with the square–star-shaped leaf-like BiVO_4_ sample at 150 °C exhibited the highest response to NH_3_ (5 ppm), with a response value of 13.3. In contrast, the responses to CO (10 ppm) and N_2_ (100 ppm) were relatively lower, at 11.1 and 10.9, respectively, while the responses to other gases were significantly lower, particularly to NO_2_ (100 ppm), methanal (10 ppm), toluene (100 ppm), acetone (100 ppm), ethanol (100 ppm), and H_2_ (10 ppm). This differentiated response indicates that the BiVO_4_ gas sensor has excellent selectivity, making it particularly suitable for NH_3_ detection. 

To verify the stability of the sensor made with the square–star-shaped leaf-like BiVO_4_ sample prepared at 150 °C, repeatability tests were conducted in a 5 ppm NH_3_ environment at 300 °C. The results are shown in Figure 8b. The response curves from multiple tests displayed almost identical peak heights, with response currents consistently observed around 38 nA, demonstrating the sensor’s excellent stability in detecting the target gas under the same conditions. This characteristic is crucial for continuous NH_3_ monitoring in practical applications. Figure 8c illustrates the transient response of the sensor prepared with the square–star-shaped leaf-like BiVO_4_ sample at 150 °C to different NH_3_ concentrations (ranging from 0.1 ppm to 5 ppm) at 300 °C. It can be observed that the response current gradually increases with the NH_3_ concentration, from 27.8 nA at 0.1 ppm to 38.7 nA at 5 ppm, showing good concentration dependency without any signs of saturation. This indicates that the sensor can sensitively respond to changes in NH_3_ concentration, facilitating accurate NH_3_ level measurements.

**Figure 9:**
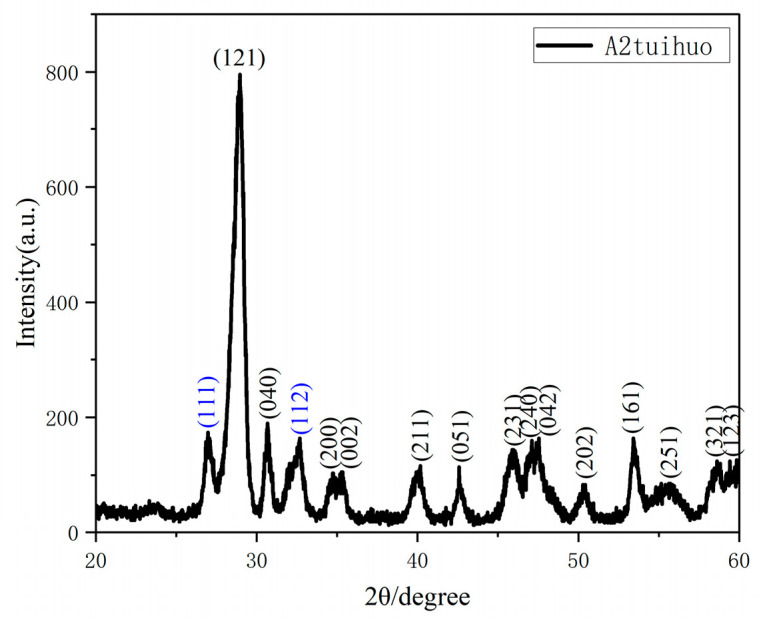
XRD pattern of the square–star-shaped leaf-like BiVO_4_ sample prepared at 150 °C after annealing.

Figure 8d presents the response and recovery times of the sensor prepared with the square–star-shaped leaf-like BiVO_4_ sample at 150 °C, which are 28 s and 16 s, respectively (The arrows in the graph indicate the time range between the start and end of the response). The fast response and recovery times indicate that the sensor has high agility in detecting NH_3_ concentration changes, enabling accurate gas concentration detection within a short period. This feature highlights the sensor’s significant potential for applications in rapid gas monitoring. Figure 8e illustrates the response of a sensor fabricated with tetragonal square–star-shaped leaf-like BiVO_4_ prepared at 150 °C to 5 ppm NH_3_ at different temperatures. It can be observed that the gas response value of the sensor gradually increases with the rise in temperature, reaching a peak value of 13.36 at 300 °C. However, when the temperature continues to increase to 350 °C, the gas response starts to decrease, indicating that 300 °C is the optimal operating temperature for the sensor. Under these conditions, the sensitivity of the sensor to NH_3_ is maximized, which provides important guidance for optimizing the operating parameters of the sensor and improving its detection performance. To evaluate the stability of the BiVO_4_ nanopowder material at 300 °C, the square–star-shaped leaf-like BiVO_4_ material prepared at 150 °C was annealed. After annealing at 500 °C for 120 min, its XRD pattern was tested and analyzed. The results showed that the diffraction peaks of the annealed sample at planes such as (121), (040), (200), (002), (211), (051), (231), (240), (042), (202), (161), (321), and (123) corresponded to the standard JCPDS card No. 14-0688, indicating that the annealed sample still retained the monoclinic scheelite structure of BiVO_4_. Among these, the (112) plane, marked in blue, corresponds to the standard JCPDS card No. 14-0133 and remains part of the monoclinic scheelite structure [42]. This demonstrates that the XRD pattern of the sample after annealing shows no significant crystal phase changes, no formation of impurity phases, and negligible changes in grain size. It is confirmed that the square–star-shaped leaf-like BiVO_4_ nanomaterials prepared by us have good structural and chemical stability at 500 °C and are suitable for long-term use. The XRD pattern is shown in Figure 9 below.

In summary, Figure 8 and Figure 9 demonstrate that the sensor fabricated from the square–star-shaped leaf-like BiVO_4_ sample prepared at 150 °C exhibits excellent selectivity, repeatability, and fast response and recovery times. It shows high sensitivity to NH_3_, with optimal performance achieved at 300 °C. 

In addition, the NH_3_ gas-sensing performance of the tetragonal square–star-shaped leaf-like BiVO_4_-based gas sensor prepared in this study was compared with that of other sensors. The comparison is shown in Table 3 below [43,44,45,46,47].

Based on the table, the square–star-shaped leaf-like BiVO_4_-based gas sensor prepared in this study shows the best response to ammonia gas, outperforming other ammonia gas sensors, and also exhibits relatively low response and recovery times. 

### 3.3. Gas-Sensing Mechanisms

The gas-sensing mechanism of the BiVO_4_ sensor primarily relies on Redox reactions between gas molecules and the material’s surface. When gas molecules come into contact with the BiVO_4_ surface, oxygen molecules adsorb onto it, capturing electrons from the conduction band of BiVO_4_ to form oxygen ions (O^−^, O_2_^−^). This process thins the electron depletion layer on the BiVO_4_ surface, changing its resistance and causing the current to change. For reducing gases (such as CO, H_2_, NH_3_, etc.), they react with the adsorbed oxygen ions on the surface, reducing them and releasing electrons back into the conduction band of the sensor, thereby reducing the resistance, increasing the current and thus improving the conductivity. The semiconductor properties of BiVO_4_ make its current highly sensitive to changes in surface electron concentration, allowing for the detection of different gases and their concentrations by monitoring current changes [48,49,50].

The gas-sensing mechanism for ammonia (NH_3_) of the BiVO_4_ gas sensor primarily relies on the characteristics of ammonia as a reducing gas and its reaction with oxygen ions on the BiVO_4_ surface [51]. The specific mechanism is as follows: When ammonia comes into contact with the BiVO_4_ gas sensor surface, NH_3_ molecules are first adsorbed on the sensor surface. BiVO_4_, as an n-type semiconductor material, adsorbs oxygen from the air on its surface, forming negatively charged oxygen ions (such as O^−^ and O_2_^−^). Ammonia, being a reducing gas, undergoes a reduction reaction with these adsorbed oxygen ions, releasing electrons back into the conduction band of BiVO_4_, thereby reducing the resistance of the sensor and raising the current to improve its conductivity [52]. This reaction can be represented as follows:

O_2_+e^−^→O_2_^−^ (2)

4NH_3_+3O_2_^-^→2N_2_+6H_2_O+6e^−^ (3)

**Figure 10:**
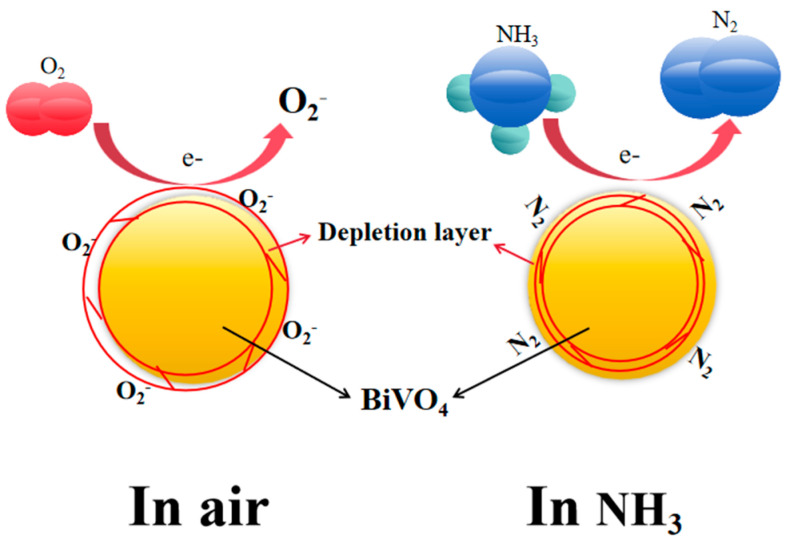
Schematic diagram of the gas-sensing mechanism of BiVO_4_ material.

In this reaction, ammonia molecules react with the adsorbed oxygen ions, producing nitrogen and water while releasing electrons. The released electrons re-enter the BiVO_4_ conduction band, causing the resistance of the sensor to decrease and the current to increase. Its mechanism is shown in Figure 10 below.

Moreover, during the reaction between NH_3_ and oxygen ions on the BiVO_4_ material surface, intermediate products such as NO and NO_2_ may be present. Relevant studies suggest that the generation of these intermediates may affect the distribution of oxygen vacancies and the thickness of the surface electron depletion layer, thereby altering the gas-sensing response. Although this study has not directly verified these intermediates through in situ surface analysis methods, the changes in the XPS oxygen vacancy peaks combined with related literature suggest that oxygen vacancies play a critical role in the NH_3_ response of the BiVO_4_ sensor by providing active sites, regulating the surface electron depletion layer, and promoting the generation and consumption of intermediates. The presence of oxygen vacancies directly enhances the sensitivity, response speed, and stability of the sensor while also optimizing the dynamic regulation of intermediate products [53].

According to related literature, significant efforts have been made to improve the anti-interference performance of NH_3_ sensors against NO_x_. For instance, using suitable sensing electrodes (SEs) such as TiO_2_ and Ni_3_V_2_O_8_ has demonstrated high selectivity toward NH_3_ with minimal response to NO_x_. The modification of SEs with secondary phases (e.g., Ag, Au) can significantly suppress NO_x_ adsorption and improve sensor selectivity. Additionally, selecting appropriate reference electrodes (REs) with moderate sensitivity to NO_x_ can counteract the response of SEs, thereby mitigating NO_x_ interference in the sensor [54,55].

In summary, the gas-sensing mechanism of ammonia with the BiVO_4_ gas sensor is mainly based on the reaction between ammonia molecules and oxygen species on the BiVO_4_ surface. This reaction releases electrons and reduces the resistance of the sensor, causing the current of the sensor to rise, thereby determining the presence and concentration of ammonia by monitoring the change in current. 

## 4. Conclusions

In summary, this study successfully prepared BiVO_4_ nanopowder materials with a square–star-shaped leaf-like structure via a hydrothermal method and systematically investigated their gas-sensing performance. The characterization of their microstructure revealed that the square–star-shaped leaf-like structure significantly increased the specific surface area, thereby enhancing gas-sensing performance. Experimental data demonstrated that the sensor fabricated from the BiVO_4_ sample prepared at 150 °C exhibited excellent selectivity, sensitivity, and response speed. At the optimal operating temperature of 300 °C, the sensitivity to 5 ppm NH_3_ reached 13.3, with response and recovery times of 28 s and 16 s, respectively. Additionally, interference tests showed that the sensor could reliably detect NH_3_ even in the presence of various interfering gases, with minimal interference from those gases. Therefore, the BiVO_4_-based gas sensor not only demonstrated high sensitivity and a rapid response capability for low-concentration ammonia but also maintained reliable operation in complex gas environments. This makes it highly suitable for applications in environmental monitoring and industrial safety detection. 

## Figures and Tables

**Table 1:** Elemental composition ratio of BiVO_4_ materials with square–star-shaped leaf-like structure prepared at 150 °C.

Element	Atomic %
O	77.1
Bi	12.5
V	10.4
Total	100.00

**Table 2:** BET specific surface areas of different samples.

Sample	S_BET_ (m^2^/g)	Average Pore size (nm)	Pore Volume (cm^3^/g)
A2 (150 °C)	2.84	17.76	0.026
A4 (170 °C)	1.21	42.09	0.021

**Table 3:** Comparison of the gas-sensitive properties of ammonia sensors made of different materials.

Material	Operating Temperature (°C)	Response Value	Response Time (s)	Recovery Time (s)	References
Pd-ZnO thin film	230	3.9	23.2	271.8	[43]
Mo-SnO_2_ nanoparticles	350	3.1	21	31	[44]
ZnO/CuO heterostructure	Room temperature	4.5	2.3	2.1	[45]
Ce-doped SnO_2_	Room temperature	6.1	4.4	12.8	[46]
Square–star-shaped leaf-like BiVO_4_	300	13.3	28	16	This research
CeO_2_/MXene	Room temperature	3.68	12	19	[47]

## Data Availability

The original contributions presented in the study are included in the article, and further inquiries can be directed to the corresponding author.
